# Predicting change in quality of life from age 79 to 90 in the Lothian Birth Cohort 1921

**DOI:** 10.1007/s11136-018-2056-4

**Published:** 2018-11-23

**Authors:** Caroline E. Brett, Dominika Dykiert, John M. Starr, Ian J. Deary

**Affiliations:** 1 Natural Sciences and Psychology, Liverpool John Moores University, Byrom Street, Liverpool, L3 3AF England UK; 2 Centre for Cognitive Ageing and Cognitive Epidemiology, University of Edinburgh, Edinburgh, UK; 3 Anna Freud National Centre for Children and Families and University College London, London, UK; 4 Alzheimer Scotland Dementia Research Centre, University of Edinburgh, Edinburgh, UK; 5 Department of Psychology, University of Edinburgh, Edinburgh, UK

**Keywords:** Quality of life, Functional status, Health, Older adults, Ageing, Mood, Functional decline

## Abstract

**Purpose:**

Quality of life (QoL) decreases in very old age, and is strongly related to health outcomes and mortality. Understanding the predictors of QoL and change in QoL amongst the oldest old may suggest potential targets for intervention. This study investigated change in QoL from age 79 to 90 years in a group of older adults in Scotland, and identified potential predictors of that change.

**Method:**

Participants were members of the Lothian Birth Cohort 1921 who attended clinic visits at age 79 (*n* = 554) and 90 (*n* = 129). Measures at both time points included QoL (WHOQOL-BREF: four domains and two single items), anxiety and depression, objective health, functional ability, self-rated health, loneliness, and personality.

**Results:**

Mean QoL declined from age 79 to 90. Participants returning at 90 had scored significantly higher at 79 on most QoL measures, and exhibited better objective health and functional ability, and lower anxiety and depression than non-returners. Hierarchical multiple regression models accounted for 20.3–56.3% of the variance in QoL at age 90. Baseline QoL was the strongest predictor of domain scores (20.3–35.6% variance explained), suggesting that individual differences in QoL judgements remain largely stable. Additional predictors varied by the QoL domain and included self-rated health, loneliness, and functional and mood decline between age 79 and 90 years.

**Conclusions:**

This study has identified potential targets for interventions to improve QoL in the oldest old. Further research should address causal pathways between QoL and functional and mood decline, perceived health and loneliness.

**Electronic supplementary material:**

The online version of this article (10.1007/s11136-018-2056-4) contains supplementary material, which is available to authorized users.

## Introduction

Older age brings increasing vulnerability as a result of physical and functional decline, and concomitant burdens on health and social care services. Maintaining good health and wellbeing are often portrayed as markers of healthy or successful ageing [1–4] and are a target for policymakers and health professionals alike [4].

Defined as “individuals’ perceptions of their position in life in the context of the culture and value systems in which they live and in relation to their goals, expectations, standards and concerns” [5], quality of life (QoL) has been characterised as a global aggregated measure, incorporating both objective and subjective indicators. As such, it is thought to be more influenced by situational factors such as material or social circumstances than measures of subjective wellbeing, such as life satisfaction or positive affect, which are more strongly associated with psychological factors [6–8]. Poor QoL has been shown to predict a range of negative health outcomes, including mortality [9–13]. Consequently, QoL is increasingly regarded as an important outcome measure for interventions aimed at improving health outcomes or reducing health inequalities [14].

The relationship between age and subjective ratings of wellbeing and QoL is complex [15, 16]. Research generally suggests a U-shaped relationship across the life course [17–20], with a decline in QoL and wellbeing amongst the oldest old aged 75 years and over [18, 21, 22]. This relationship appears to differ depending on the measure used. Growing evidence suggests that, when measured in terms of life satisfaction, wellbeing is relatively stable even amongst the oldest old and ‘bounces back’ following negative life events (including spousal death) to a set point [23–25], which itself is determined largely by psychological factors developed over the life course [26, 27]. In contrast, QoL appears to fluctuate over time, with individual trajectories determined predominantly by changing circumstances rather than age [18, 28, 29].

Understanding the predictors of individual differences in both the current level of and change in QoL over time amongst older adults is important for identifying potential targets for intervention. Although the majority of research to date has been cross-sectional, it has identified a number of strong candidates for potential predictors of QoL in old age, including functional status (activities of daily living (ADLs)) [18, 30–32], health (predominantly chronic conditions) [30, 31, 33], depression [20, 30, 33–37], anxiety [14, 30], marital status [18, 28, 31], quality of social contacts [30, 38], socioeconomic conditions [31, 38], and personality [30, 37]. Longitudinal studies have suggested that current circumstances influence QoL judgements more than early life circumstances [39], which might exert influence on QoL indirectly through current circumstances such as property ownership and health [40]. Using data from the Berlin Aging Study, Baltes and colleagues identified a psychological profile amongst their oldest participants with the highest wellbeing which included many of the above factors [41].

Studies investigating predictors of change in QoL in older age are scarce, especially amongst the oldest old. Webb et al. [28] showed that QoL decreased over 4 years amongst over-50s, that decline was associated with increased depression and difficulties with ADLs, but that improvements in family relationships, neighbourhood, and perceived financial position counteracted decline in QoL. Chan et al. [33] found that increased physical illness, depressive symptoms, and difficulties with instrumental ADLs, were all associated with decreased QoL over 12 months in over-65s diagnosed with a depressive disorder. Some researchers suggest that, over time, older adults place increasing emphasis on health and mobility when determining their QoL [30, 39]. With growing numbers of people reaching their ninth decade and experiencing declining physical and cognitive capabilities, investigation of how QoL changes during this time is timely and essential [42].

The current study aims to investigate change in QoL between the ages of 79 and 90 years in a group of older adults in Scotland, and to identify potential predictors of that change. We have previously shown that depression, emotional stability, conscientiousness, social class, living alone, and health had significant cross-sectional associations with QoL in this Lothian Birth Cohort 1921 (LBC1921) group at age 79, and that determinants differed between QoL domains [37]. In the present study, we hypothesised that mean QoL would decline from age 79 to age 90 and that baseline QoL would be the strongest predictor of current QoL. Based on our own and previous research, we also hypothesised that increases (age 79–90) in depression, changes in living-alone status, health, and functional status, personality, and occupational social class would all be associated with changes in QoL.

## Methods

### Participants

The participants were all members of the LBC1921, which has been described in detail elsewhere [43]. The LBC1921 Study consists of 550 individuals (238 men), most of whom participated in the Scottish Mental Survey 1932 aged around 11 years [44]. Between 1999 and 2001, participants—all of whom were living independently and aged around 79 years—undertook detailed cognitive and physical testing, and answered questions related to their health, occupation, and lifestyle. At age 80–81, 497 participants completed a questionnaire which included QoL items, and, at age 81, 467 participants completed a personality questionnaire. Additional waves of follow-up testing were completed at age 83 and 87 [43].

In 2011, at age 90 years, all participants, except those who had died (*n* = 190, 34.5%), withdrawn (*n* = 73, 13.3%), lost contact (*n* = 15, 2.7%), were not well enough, or were ineligible to participate (*n* = 105, 19.1%; this included participants with a diagnosis of dementia), or were unable to take part for other reasons (*n* = 27, 4.9%; moved away or had caring responsibilities), were invited to a fourth wave of follow-up testing [45]. Participants with dementia were excluded due to the study’s emphasis on non-pathological cognitive ageing; sensitivity analyses have shown that incipient dementia had little influence on key findings from this cohort [46]. 129 participants attended a clinic visit involving comprehensive cognitive and physical testing and a structured interview, as per wave 1. They also completed an extensive questionnaire, which included a repeat of the QoL items. The current study uses data from wave 1 and wave  4 clinic visits, and the QoL and personality questionnaires completed at age 80/81.

### Measures

#### Repeated measures

##### Quality of life

QoL was measured at waves 1 and 4 (ages 79 and 90) using the WHOQOL-BREF, which is a well-validated abbreviated version of the WHOQOL-100 quality of life assessment containing 26 items [47–50]. The WHOQOL-BREF contains one item from each of the 24 facets of QoL included in the WHOQOL-100 and which produce scores on four QoL domains: physical (7 items, e.g. “How satisfied are you with your sleep?”), psychological (8 items, e.g. “To what extent do you feel your life to be meaningful?”), social (3 items, e.g. “How satisfied are you with your personal relationships?”), and environment (9 items, e.g. “How satisfied are you with the conditions of your living place?”), and two additional items measuring overall QoL and general health. Participants are asked to consider the extent to which each item reflects their experiences over the last 2 weeks, and indicate their response on a 5-point Likert-type scale. As per the WHOQOL-BREF manual [48], domain scores were calculated by multiplying the mean score across all items relating to that domain by 4, resulting in a score out of 20. The single items were scored out of 5. In all cases, higher scores indicated better QoL. For the purposes of correlational and ordinal regression analysis, the single-item scores were collapsed into either three (poor/neither poor nor good, good, very good) or four (very/dissatisfied, neither, satisfied, very satisfied) categories for the QoL and health QoL items, respectively.

As reported elsewhere [37], one question (q21: “How satisfied are you with your sex life?”) was judged to be inappropriate for this age group and reworded as “How satisfied are you with the support you get from your family?” Missing values were handled using a pro-rating technique, whereby single missing values in each domain were replaced with the mean of the remaining items from that domain. Where multiple values were missing in a domain, these were not replaced. Cronbach’s alphas indicated acceptable to good internal consistency for all domains at both waves (wave 1: *α* = 0.730 to 0.844; wave 4: *α* = 0.617 to 0.751).

Scores on the four domains and two single items at wave 1 were subtracted from their wave 4 counterparts in order to calculate raw change in QoL over time, with positive values indicating increased QoL.

##### Current mood

The Hospital Anxiety and Depression Scale (HADS) [48] was used to measure symptoms of anxiety and depression at waves 1 and 4. The HADS consists of 7 items each for anxiety and depression, each scored on a 3-point scale giving a maximum score of 21. Scores of 8 + are seen to suggest possible cases of anxiety disorders or depression [51, 52].

##### Functional ability

The Townsend Functional Ability Scale [53] was used to measure current functional status at waves 1 and 4. Participants were asked how able they were to complete a series of nine everyday tasks. Each was scored on a 3-point scale with a maximum score of 18; higher scores indicated greater impairment.

##### Objective health status

Biomarkers are considered key indicators of healthy ageing [54, 55], and are more reliable than self-report measures of health or health behaviours [56].

Grip strength, peak expiratory flow (PEF), and Body Mass Index (BMI) were used as objective measures of health status. All were measured during the clinic visit at waves 1 and 4. Grip strength has been shown to be related to future health outcomes [57] and cognition [58] amongst older adults. This was measured using a dynamometer; three measurements were taken for each hand and the best of all six used in the analyses. PEF has been shown to be a valid index of health status in older adults and independently associated with ADLs, hospitalisation, and subjective mortality risk assessment [59, 60]. This was measured using a spirometer. Participants were asked to blow into the apparatus for as long as they could; the best of three measurements was taken. Both variables were adjusted for sex and height at the time of testing and the standardised residuals used in subsequent analyses. High and low BMI has been associated with lower health-related QoL and physical functioning in older adults [61]. Height and weight were measured during each clinic visit.

Residualised change scores for the mood, functional ability, and objective health measures were calculated by regressing equivalent wave 4 scores on wave 1 (baseline) scores. In accordance with the directionality of the original measures, higher change scores indicate worsening anxiety, depression, and functional ability, and less decline in (or relatively improving) grip strength and lung function.

##### Living-alone change

Participants were asked at both waves whether they lived alone or not. This was converted into a change score based on the anticipated impact of the change from wave 1 to wave 4, as follows: − 1 = change from not alone to alone (*n* = 32), 0 = no change (*n* = 90), 1 = change from alone to not alone (*n* = 6).

#### Baseline (wave 1; age 79) measures

##### Social class

Participants were asked at wave 1 to provide details of their highest status occupation. Using the 1951 Classification of Occupations [62], this was used to derive their occupational social class within five groupings: I (professional), II (managerial and technical), III (skilled), IV (semi-skilled), and V (unskilled). Female participants were also asked for their husband’s occupation (where applicable) and the higher of the two was used to represent their social class. Social class was deemed to have both a distal and proximal influence on QoL through its effect on income and therefore material circumstances in old age.

##### Personality

The 50-item version of the International Personality Item Pool (IPIP) [63–65] was used at wave 1 to measure scores on the Big Five personality traits of extraversion, agreeableness, conscientiousness, emotional stability, and intellect/imagination. Scores for each trait were calculated by summing responses to 10 items placed on a 5-point Likert-type scale. For the present analysis, only those traits that previous research has shown to be strongly associated with QoL [37, 66] were included, i.e. conscientiousness and emotional stability (the inverse of neuroticism).

##### Loneliness

Loneliness was measured at wave 1 using a single item, “At the present moment do you feel lonely?”, answered from a choice of five options (scored 5–1, higher scores indicating higher loneliness): most of the time, quite often, only occasionally, seldom, and never.

#### Current (wave 4; age 90) measures

##### Self-rated health

Self-rated health was measured at wave 4 using a single item: “How would you rate your health just now?”, answered from five options (scored 5–1): excellent, very good, good, fair, and poor.

### Statistical analysis

All statistical analyses were carried out using SPSS Statistics for Windows, Version 24.0 (IBM Corp, Armonk NY).

Independent sample *t* tests were conducted to compare participants who returned for wave 4 with those who did not on all baseline measures. Paired *t* tests were then conducted to identify significant differences between all repeated measures from baseline and wave 4.

As all variables were found to be non-normally distributed, Spearman’s rho bivariate correlations were calculated between all baseline and change variables, and the QoL measures at both waves. To correct for multiple testing, the false discovery rate (FDR) was controlled (*α* = 0.05) using a procedure described in Benyamini and Hochberg [67]. To enhance model parsimony, only variables with correlations that were significantly associated with the relevant QoL measure below the critical *p* value identified by the FDR calculation were included in subsequent regression analyses.

A series of hierarchical multiple linear regression analyses were carried out with each of the four QoL domains as the outcome variable. In order to test the additional contribution made by each category of predictor variable, baseline scores on the QoL measure were included at the first step, baseline predictors (including personality) at the second step, change variables (functional ability and mood) at the third step, and current measures at the fourth step. Finally, proportional odds logit ordinal regression was carried out with the two single QoL items as the outcome variables and variables at each level (baseline QoL, other baseline, change, current) added in turn. Again, FDR calculations were carried out for each analysis type to control for multiple testing.

## Results

Descriptive statistics for the QoL and predictor variables at both waves of testing are shown in Table 1. For wave 1, these are shown for all participants and by returners versus non-returners. Participants who did not return for wave 4 scored significantly lower on physical, psychological, and environmental QoL, and on the two single QoL items at wave 1, as well as reporting significantly higher anxiety, depression, and functional limitations, scoring significantly lower on emotional stability, and exhibiting lower grip strength and lung function (these differences were only statistically significant in women and men, respectively).

**Table 1:** Means, etc. for WHOQOL-BREF domains and items, and all predictor variables for both waves

	All w1		W1 non-returned	W1 returned^a^	W4		Change (w1–w4)
	Mean (SD)	*α*	Mean (SD)	Mean (SD)	Mean (SD)	*α*	Mean (SD)
*N*	460–554		325–425	129	124–129		123–129
Physical QoL	14.86 (2.78)	0.844	14.64 (2.84)	15.62** (2.37)	13.78 (2.51)	0.751	− 1.89*** (2.23)
Psychological QoL	15.28 (2.04)	0.764	15.12 (2.18)	15.74** (1.55)	14.49 (2.00)	0.748	− 1.24*** (1.63)
Social QoL	17.28 (2.36)	0.730	17.15 (2.46)	17.61 (2.00)	17.51 (2.30)	0.739	− 0.07 (2.26)
Environment QoL	16.48 (2.10)	0.785	16.27 (2.18)	17.06*** (1.67)	15.94 (1.64)	0.617	− 1.09*** (1.65)
QoL item	4.28 (0.71)	–	4.21 (0.73)	4.46*** (0.60)	3.98 (0.70)	–	− 0.49*** (0.74)
QoL health item	3.67 (0.93)	–	3.58 (0.94)	3.98*** (0.81)	3.44 (0.96)	–	− 0.56*** (0.91)
Townsend	2.26 (2.80)	0.783	2.51 (2.98)	1.44 *** (1.87)	5.10 (4.56)	±	3.66*** (3.98)
Grip strength: men	26.74 (8.97)	–	26.40 (9.03)	27.93 (8.75)	20.71 (8.18)	–	− 7.30*** (5.13)
Grip strength: women	26.39 (9.23)	–	25.48 (8.89)	29.19** (9.72)	21.10 (8.43)	–	− 8.15*** (4.73)
PEF: men	277.84 (140.00)	–	265.90 (136.92)	320.10* (143.98)	243.84 (121.71)	–	− 78.82*** (104.89)
PEF: women	267.80 (126.96)	–	260.32 (126.05)	290.69 (127.79)	220.32 (97.15)	–	− 70.63*** (91.24)
Anxiety	5.20 (3.31)	0.758	5.42 (3.43)	4.45** (2.72)	3.71 (2.87)	0.762	− 0.69** (2.34)
Depression	3.53 (2.33)	0.614	3.69 (2.40)	3.01** (1.97)	3.82 (2.35)	0.521	0.86*** (2.32)
Living alone (%)	48.0%	–	48.3%	46.9%	66.7%	–	
IPIP C	38.71 (6.08)	0.766	38.63 (6.09)	38.96 (6.05)	–	–	–
IPIP ES	34.24 (8.13)	0.872	33.53 (8.20)	36.37** (7.59)	–	–	–
Loneliness	1.97 (1.02)	–	2.01 (1.05)	1.83 (0.93)	–	–	–
Social class	2.24 (0.88)	–	2.30 (0.87)	2.04 (0.89)		–	
Self-rated health	–	–	–	–	3.56 (0.87)	–	–

For W1 returned column, p values reflect differences between returners and non-returners. For change column, p values reflect differences between wave 1 and wave 4. ± Item-level data were not available for the Townsend Functional Ability Scale at wave 4

*t Test *p* < .05; **t test *p* < .01; ***t test *p* < .001

^a^These are individuals who attended both wave 1 and returned for wave 4

**Fig. 1:**
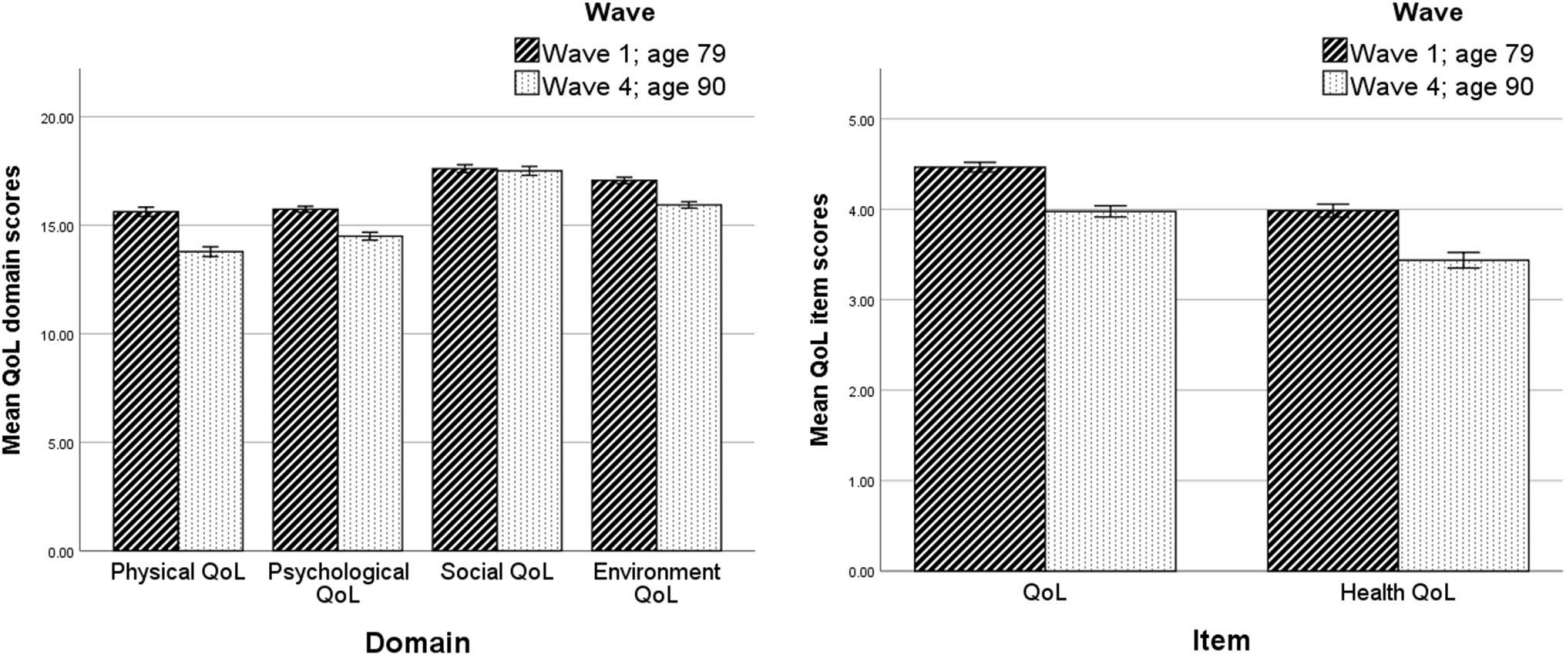
Mean scores on QoL domains and single QoL items for returners at age 79 and 90 (error bars represent standard errors)

Participants who completed both waves scored significantly lower at age 90 than they had done at age 79 on all QoL measures except the social domain (see Table 1; Fig. 1). They also reported significantly more functional limitations, lower anxiety, and higher depression, and exhibited significantly lower grip strength and lung function at age 90 than they had done at age 79.

Table 2 contains the results of Spearman’s bivariate correlations between the six QoL measures from wave 4 and all the predictor variables, including baseline QoL. Most of the strongest associations were between the comparable QoL measures at baseline (wave 1) and wave 4, with rho values ranging from 0.36 (QoL item) to 0.61 (psychological QoL). Higher physical QoL and the single health QoL item were both associated with lower age-79–90 reduction in and higher current functional ability, lower age-79–90 increase in and lower current depression, and higher current self-rated health. Higher physical QoL was also associated with higher current grip strength. Higher psychological QoL was associated with higher baseline emotional stability, lower baseline loneliness, lower age-79–90 increase in and lower current anxiety and depression, and higher current self-rated health. Social QoL was not significantly associated with any predictor variables. Higher environmental QoL was associated with higher emotional stability and occupational social class, lower baseline loneliness, lower current anxiety and depression, and higher current self-rated health. The single QoL item was associated with lower baseline loneliness, lower age-79–90 reduction in and higher current functional ability, lower age-79–90 increase in and current depression, and higher current self-rated health and lung function.

**Table 2:** Correlations between QoL measures at wave 4 and all predictor variables

W4 QoL	Baseline (wave 1, age 79)												Change (age 79–90)**							Current (wave 4, age 90)						
	QoL Physical	QoL Psych	QoL Social	QoL Envir	QoL item	QoL health	Age @w1	Sex	Social class	IPIP C	IPIP ES	Loneliness	ADL change	Grip change	PEF change	BMI change	Anx. change	Dep change	Living change	ADL	Anxiety	Depression	Grip	PEF	BMI	SR health
QoL physical	0.56*	0.29*	0.13	0.21	0.33*	0.44*	0.05	0.01	− 0.02	0.13	0.12	− 0.07	− 0.46*	0.13	0.04	− 0.19	− 0.01	− 0.47*	0.08	− 0.62*	− 0.14	− 0.55*	0.26*	0.13	− 0.28*	0.49*
QoL psych	0.42*	0.61*	0.43*	0.26*	0.39*	0.38*	0.16	0.06	0.10	0.22	0.26*	− 0.36*	− 0.18	0.06	0.17	− 0.09	− 0.30*	− 0.27*	0.08	− 0.20	− 0.41*	− 0.41*	0.13	0.13	− 0.03	0.45*
QoL social	0.27*	0.32*	0.48*	0.22*	0.15	0.22*	− 0.11	0.06	− 0.02	0.06	0.09	− 0.18	0.06	− 0.01	0.21	0.11	− 0.04	− 0.06	− 0.03	− 0.02	− 0.11	− 0.16	− 0.02	0.15	0.02	0.12
QoL envir	0.39*	0.34*	0.33*	0.49*	0.28*	0.30*	0.09	0.06	− 0.28*	0.16	0.21*	− 0.25*	− 0.19	0.09	− 0.01	0.16	− 0.15	− 0.20	− 0.02	− 0.26*	− 0.28*	− 0.32*	0.07	− 0.03	0.14	0.23*
QoL item	0.32*	0.40*	0.33*	0.30*	0.36*	0.37*	0.15	− 0.02	− 0.05	0.10	0.13	− 0.37*	− 0.26*	0.15	0.12	− 0.08	− 0.09	− 0.28*	0.05	− 0.32*	− 0.15	− 0.36*	0.20	0.25*	− 0.06	0.43*
Health QoL item	0.28*	0.22*	0.14	− 0.02	0.24*	0.49*	0.08	− 0.07	0.15	0.07	0.07	− 0.03	− 0.23*	0.12	0.04	− 0.13	0.03	− 0.26*	0.11	− 0.25*	0.02	− 0.27*	0.17	0.05	− 0.17	0.58*

*n* = 93–125

*QoL* quality of life, *Psych* psychological, *Envir* environmental, *ADL* activities of daily living (functional ability), *PEF* peak expiratory flow, *IPIP C* conscientiousness, *IPIP ES* emotional stability, *Anx* anxiety, *Dep* depression, *SR* self-rated

*Significant after FDR calculations (approximately *p* < .02)

**Higher change scores indicate worsening functional ability, anxiety, and depression, but less decline in (or improving) grip strength and lung function, and movement from living alone to not alone

The results of the final models from the hierarchical regression analyses for each QoL domain are in Table 3. The models account for between 20.3% (social QoL) and 56.3% (physical QoL) of the variance in the QoL measures. For all the domain scores, the strongest predictor was baseline QoL in the given domain, accounting for the majority of the variance explained. Physical QoL was also significantly predicted by age-79–90 change in functional ability and depression. Psychological QoL was significantly predicted by age-79–90 change in anxiety, and current self-rated health. Social QoL and environmental QoL were only significantly predicted by the baseline scores. Supplementary Tables S1–S4 show the results for each step of the hierarchical linear regression analyses. Overall, baseline QoL explained between 20.3 and 35.6% of the variance in the equivalent current QoL measures, other baseline predictors contributed between 0.4 and 3.1% additional variance, age 79–90 change variables contributed between 8.3 and 23.3%, and current predictors contributed between 2.3 and 9.2%.

**Table 3:** Results of the final model of the hierarchical multiple linear regression for the QoL domains

Step	Predictor	Physical QoL (*n* = 92)					Psychological QoL (*n* = 120)					Social QoL (*n* = 123)					Environment QoL (*n* = 120)				
		*B*	SE	95% CI for *B*	*β*	*p*	*B*	SE	95% CI for *B*	*β*	*p*	*B*	SE	95% CI for *B*	*β*	*p*	*B*	SE	95% CI for *B*	*β*	*p*
1	Baseline QoL	**0.45**	**0.07**	**0.31–0.60**	**0.45**	< .**001**	**0.48**	**0.11**	**0.26–0.71**	**0.38**	< .**001**	**0.52**	**0.09**	**0.34–0.71**	**0.46**	< .**001**	**0.32**	**0.09**	**0.14–0.51**	**0.33**	.**001**
2	Loneliness	–	–	–	–	–	− 0.25	0.18	− 0.11–0.61	− 0.11	.169	–	–	–	–	–	− 0.16	0.16	− 0.15–0.47	− 0.09	.313
	IPIP ES	–	–	–	–	–	0.01	0.02	− 0.02–0.05	0.05	.467	–	–	–	–	–	0.02	0.02	− 0.02–0.06	0.09	.293
	Social class	–	–	–	–	–	–	–	–	–	–	–	–	–	–	–	− 0.25	0.16	− 0.56–0.06	− 0.14	.119
3	ADL chg	**− 0.64**	**0.20**	**− 1.04 to −0.23**	**− 0.23**	.**002**	–	–	–	–	–	–	–	–	–	–	–	–	–	–	–
	Depression chg	**− 0.67**	**0.19**	**− 1.05–0.30**	**− 0.27**	.**001**	− 0.26	0.15	− 0.56–0.04	− 0.13	.083	–	–	–	–	–	–	–	–	–	–
	Anxiety chg	–	–	–	–	–	**− 0.42**	**0.14**	**− 0.70 to − 0.15**	**− 0.21**	.**002**	–	–	–	–	–	–	–	–	–	–
4	ADL w4	–	–	–	–	–	–	–	–	–	–	–	–	–	–	–	− 0.05	0.03	− 0.11–0.01	− 0.14	.098
	Anxiety	–	–	–	–	–	–	–	–	–	–	–	–	–	–	–	− 0.06	0.05	− 0.16–0.04	− 0.10	.245
	Depression	–	–	–	–	–	–	–	–	–	–	–	–	–	–	–	− 0.06	0.06	− 0.19–0.06	− 0.09	.331
	Grip strength	0.24	0.18	− 0.11–0.59	0.10	.178	–	–	–	–	–	–	–	–	–	–	–	–	–	–	–
	BMI	**− 0.12**	**0.05**	**− 0.22–.02**	**− 0.17**	.**020**															
	Self-rated health	0.35	0.23	− 0.10–0.80	0.12	.123	**0.58**	**0.17**	**0.25–0.92**	**0.26**	.**001**	–	–	–	–	–	0.31	0.16	− 0.00–0.61	0.16	.050
Adjusted *r*^2^		.**585**					.**474**					.**203**					.**332**				

Bold indicates significant predictors after false discovery rate calculation (approx. *p* < .035). Step 1 = baseline QoL (age 79; equivalent measure to outcome); step 2 = other baseline variables (age 79); step 3 = change variables (age 79–90); step 4 = current variables (age 90)

*QoL* quality of life, *ADL* activities of daily living (measured with Townsend Functional Ability Scale), *IPIP ES* International Personality Item Pool Emotional Stability, *Chg* change, *BMI* Body Mass Index

The results of the ordinal regression analyses are shown in Table 4 and Supplementary Tables S5, S6. In the final models, the QoL item was significantly predicted by baseline loneliness and current self-rated health. The health QoL item was significantly predicted by baseline health QoL and current self-rated health. Pearson’s goodness-of-fit test indicated that for both models the observed probabilities did not deviate from the probabilities predicted by the multinomial regressions, suggesting that the models fit the data well (*χ*^2^ = 183.36, *p* = .356 and *χ*^2^ = 370.08, *p* = .293). For the QoL item, baseline QoL significantly predicted current QoL for the first two models, but this effect was attenuated when current predictors were added.

**Table 4:** Results of the final model of the proportional odds ordinal regression for the QoL and health QoL items

Predictor	QoL item					Health QoL item				
	Estimate	SE	*p*	OR	95% CI for OR	Estimate	SE	*p*	OR	95% CI for OR
Baseline QoL	0.32	0.48	.503	1.38	0.54–3.55	**0.88**	**0.26**	.**001**	**2.40**	**1.46–3.96**
Loneliness age 79	**− 0.91**	**0.31**	.**004**	**0.40**	**0.22–0.74**	–	–	–	–	–
ADL change	− 0.34	0.30	.246	0.71	0.40–1.27	− 0.35	0.20	.074	0.70	0.48–1.03
Depression change	− 0.32	0.29	.277	0.73	0.41–1.29	0.14	0.21	.509	1.15	0.76–1.73
Lung function age 90	0.30	0.25	.230	1.35	0.83–2.21	–	–	–	–	–
Self-rated health age 90	**1.05**	**0.38**	.**005**	**2.85**	**1.37–5.94**	**1.27**	**0.27**	< .**001**	**3.58**	**2.12–6.03**

Bold indicates significant predictors after false discovery rate calculation (approx. *p* < .029)

*QoL* quality of life, *ADL* activities of daily living (measured with Townsend Functional Ability Scale), *SE* standard error

## Discussion

This study is one of the first to investigate predictors of change in QoL across 11 years of old–old age. The results show that QoL declined significantly across most domains and the QoL and health single items between 79 and 90, as did functional ability and objective health status. This is in keeping with the results of previous studies [18, 22, 28, 39]. Baseline QoL, functional ability, grip strength, lung function, and emotional stability were all higher and anxiety and depression lower in returners compared to non-returners, pursuant to research suggesting that retention in longitudinal studies is higher amongst those who are advantaged [28]. Given the principal reason for attrition in the LBC1921 was mortality, poor health, or poor function [45, 68], this finding further supports the suggestion that poor QoL is associated with poorer health outcomes and mortality [9, 11–13, 69].

The findings suggest that, for the four QoL domains, QoL at age 90 is most strongly associated with QoL earlier in old age. Lower physical QoL was also associated with worsening depression and functional ability and psychological QoL with increasing anxiety. These results support previous research [18, 30]. The single QoL item was associated with baseline loneliness and current self-rated health, while the health QoL item was associated with baseline health QoL and current self-rated health.

The largest single determinant of QoL domain scores at age 90 was QoL at age 79. This supports previous research suggesting that QoL is comparatively stable within individuals in old age and ‘bounces back’ from adversity [29, 70]. Much research into healthy ageing has focussed on individual differences in psychological resources such as resilience, optimism, personality, coping, sense of coherence, and perceived social support. These are thought to influence individuals’ ability to cope and maintain their wellbeing in the face of adversity, and, crucially, their subjective experience and ratings of their health and wellbeing [41, 70]. However, QoL stability contrasts with research suggesting QoL is sensitive to changes in physical health [6]. In the present study, the final models only explained at most a third of the variance in social QoL, and environmental QoL. This suggests that factors not included in this analysis are likely to be important and more research is needed to identify them. Of note, social QoL—measured here as satisfaction with personal relationships, friends, and family—was not explained by any other factors, suggesting it is unaffected by physical and health limitations and may be a suitable target for intervention amongst the oldest old. This complements current approaches towards improving wellbeing in later life [42, 71].

In contrast to previous research [37, 40], change in living-alone status was not associated with QoL here. Loneliness at age 79 was, however, associated with scores on the single QoL item at age 90. Numerous studies have supported the view that perceived loneliness is a stronger determinant of subjective wellbeing in older adults than either marital status or living arrangement [28, 72, 73]. Longitudinal data on loneliness and marital status were not available in the LBC1921 sample, preventing investigation of their differential associations with QoL here.

The finding that increased depressive symptoms only significantly contributed to physical QoL, and no other QoL measures, contrasts with our own research [37] and that of others [36, 74]. One explanation might be that the LBC1921 participants who returned for wave 4 reported relatively few depressive symptoms, with few scoring above the threshold for possible caseness of depression and a median score of 3 out of 21. Current depressive symptoms were significantly associated with all the QoL measures except social QoL, but was excluded from most of the regression analyses due to collinearity. Rerunning the regression analyses using the stepwise entry method and including current depressive symptoms did not yield different results to those reported here. Nevertheless, minor depressive symptoms do appear to affect QoL. Indeed, Chang et al. [74] suggest that low levels of depressive symptoms are associated with similar demographic and health risks as major depression, and are clinically important as they may indicate subsyndromal depression and other mental health problems. Adverse life events including widowhood, change in financial circumstances, or the onset of disabling illness, are common in old age and can lead to increased depressive symptoms and concomitant decline in QoL. Targeting older adults at these critical points by offering support and/or boosting their internal locus of control might prevent decline in QoL [14, 34]. Health services may also benefit from greater identification of older adults who are experiencing depressive symptoms [33, 34], which may be underreported.

Functional decline, as indicated by increased impairment in ADLs, was strongly associated with physical QoL. Again, this supports previous research [18, 30–32]. Declining functional ability can lead to a loss of independence and self-confidence, with a consequent decline in mood and QoL. However, some older adults retain high QoL despite low functioning, and they tend to report greater perceived control and use of adaptive coping strategies [75, 76]. Assuming a causal association between perceived control/coping and QoL, services which improve coping abilities and overall functioning in older people—particularly those suffering from chronic illness such as arthritis—might also improve their mood and subsequent QoL [33, 75].

Objective health status, as measured by grip strength and lung function, did not significantly contribute to QoL. Self-rated health, however, significantly contributed to psychological QoL, and the two single QoL items. This is unsurprising: previous research suggests self-rated health may be strongly predictive of QoL and other health and wellbeing outcomes in older adults [77–79]. As QoL is largely evaluative, individuals’ subjective experiences of their health status may be more influential than objective measures [28].

Stable factors such as social class and personality did not significantly contribute to QoL. However, we have previously shown that these factors predicted age-79 QoL in this group [37]. Given that age-79 QoL was the strongest predictor of age-90 QoL, this suggests that the impact of these stable factors occurs at an earlier timepoint.

The finding that analyses differed by QoL domain supports the notion of QoL as a multi-faceted construct [6, 47]. However, there may be construct overlap between QoL and the predictors described here. Indeed, several WHOQOL-BREF items tap into elements of functional ability and depressive symptoms, asking about ADL, ability to get around, sleep, energy, or ability to concentrate. Nevertheless, the findings highlight the importance of small changes to functional ability, mood, and self-rated health to QoL—all of which are more salient to a carer, health professional, family member, or older person than global QoL.

### Strengths and limitations

The participants were all part of the LBC1921 cohort and living in Scotland. It is possible, therefore, that these results are subject to cohort effects and not generalisable to other countries or time periods [31]. For example, Jivraj and Nazroo [31] found that socioeconomic inequalities and education were stronger predictors of QoL in the USA than in England. However, health—measured by functional ability and chronic conditions—was the strongest predictor of QoL in both countries, supporting the present study’s findings. Nevertheless, further replication is essential to enable cohort-based research to influence policymakers or health service provision.

As with all longitudinal studies, the LBC1921 suffered from considerable attrition between waves 1 and 4. While many non-returning participants had died or were suffering from dementia-related illnesses, a significant number chose not to participate due to poor health or functional limitations [45, 68], as illustrated by the significant differences observed between returners and non-returners on relevant baseline measures. Given that functional ability and depression were important predictors of QoL, it is likely that attrition attenuated the study’s findings.

The study suffered from a lack of comparative measures between wave 1 and wave 4. For example, waves 2–4 of the LBC1921 included measures of loneliness, marital status, living conditions, optimism, resilience, and mental wellbeing. All are potential determinants of QoL, but could not contribute to the present study, which focussed on longitudinal analysis.

The small sample size did not allow for comparative analysis of the results by gender. Although men and women in this sample did not significantly differ on the QoL measures, it is possible that the pattern of associations and predictors may have been different—as noted in our previous research [37]. Future research might, therefore, test for effect modification by gender.

A further limitation—inherent to correlational research—is the strong likelihood of Type I errors. However, FDR control was used to reduce the effect of multiplicity [67].

The study’s strengths include its long follow-up and the inclusion of participants who are what is termed as the ‘oldest old’. Very few studies have included participants aged 90 or above, and most have fewer participants than this study. Furthermore, the sample’s homogeneity eliminates important confounders such as age, and geographical, cultural, and political influences.

## Conclusions and implications

Measures of subjective wellbeing, including QoL, are increasingly popular with policymakers as indicators of population health [24, 80, 81], and as a key outcome measure of social care and mental health interventions [82, 83]. The present study suggests that QoL amongst the oldest old is predominantly predicted by QoL measured earlier in the ageing process, indicating that QoL may be relatively stable in old age. This also suggests that QoL might not be a sensitive measure of short-term change. The results suggest varying patterns of association across different QoL measures, with self-rated health, functional ability, depression, and anxiety, and loneliness all contributing in varying degrees. This study contributes to an expanding body of research suggesting that worsening functional ability, depression, and anxiety, and lower current self-rated health, are strongly predictive of poor QoL in the oldest old. Future research should explore causal pathways and identify factors influencing older adults’ perception of their health, the impact of negative life events, and the psychological resources which enhance older adults’ ability to cope with the challenges inherent to the ageing process. Interventions to reduce the impact of chronic conditions and illness on subsequent function, and to improve coping and resilience amongst older adults in general, might be beneficial for the health and wellbeing of the oldest old.

## Electronic supplementary material

Below is the link to the electronic supplementary material.

Supplementary material 1 (DOCX 32 KB)
