# Supplementary material for: Predicting change in quality of life from age 79 to 90 in the Lothian Birth Cohort 1921
Source: Qual Life Res. 2018 Nov 23;28(3):737–49. doi: 10.1007/s11136-018-2056-4 (PMC6394510; doi:10.1007/s11136-018-2056-4)
Supplement: Supplementary file 1 — Supplementary material 1 (DOCX 32 KB) [file 11136_2018_2056_MOESM1_ESM.docx]

Supplementary tables

*Hierarchical regressions*

Table S1: hierarchical regression for QoL physical domain wave 4

| Category | Predictor | B | SE | 95% CI for B | β | p | B | SE | 95% CI for B | β | p | B | SE | 95% CI for B | β | p |
| --- | --- | --- | --- | --- | --- | --- | --- | --- | --- | --- | --- | --- | --- | --- | --- | --- |
| **Baseline QoL** | **Physical domain w1** | **.58** | **.09** | **.40-.75** | **.58** | **<.001** | **.53** | **.07** | **.38-.67** | **.52** | **<.001** | **.47** | **.08** | **.32-.62** | **.47** | **<.001** |
| **Change** | **ADL change** |  |  |  |  |  | **-.74** | **.20** | **-1.15--.34** | **-.27** | **<.001** | **-.67** | **.21** | **-1.10--.28** | **-.25** | **.001** |
|  | **Depression change** |  |  |  |  |  | **-.82** | **.19** | **-1.19--.45** | **-.33** | **<.001** | **-.67** | **.20** | **-1.06--.28** | **-.27** | **.001** |
| **Current** | Grip strength |  |  |  |  |  |  |  |  |  |  | .15 | .18 | -.20-.50 | .06 | .397 |
|  | Self-rated health |  |  |  |  |  |  |  |  |  |  | .44 | .23 | -.01-.90 | .16 | .057 |
|  | Constant | 5.05 | 1.38 | 2.32-7.79 |  | <.001 | 5.58 | 1.14 | 3.33-7.84 |  | <.001 | 4.90 | 1.22 | 2.49-7.32 |  |  |
| r2 change | | .331 | | | | | .233 | | | | | .023 | | | | |
| Adjusted r2 | | .323 | | | | | .549 | | | | | .563 | | | | |

B = unstandardised coefficient β = standardised coefficient ADL = Activities of Daily Living (measured by Townsend Functional Ability Scale). PEF = Peak Expiratory Flow

In bold = significant after FDR calculation.

Table S2: hierarchical regression for QoL psychological domain wave 4

| Category | Predictor | B | SE | 95% CI for B | β | p | B | SE | 95% CI for B | β | p | B | SE | 95% CI for B | β | p | B | SE | 95% CI for B | β | p |
| --- | --- | --- | --- | --- | --- | --- | --- | --- | --- | --- | --- | --- | --- | --- | --- | --- | --- | --- | --- | --- | --- |
| **Baseline QoL** | **Psych. Domain w1** | **.77** | **.09** | **.58-.95** | **.60** | **<.001** | **.71** | **.12** | **.47-.94** | **.55** | **<.001** | **.58** | **.12** | **.35-.81** | **.46** | **<.001** | **.48** | **.11** | **.26-.71** | **.38** | **<.001** |
| Baseline | IPIP ES |  |  |  |  |  | .01 | .02 | -.03-.05 | .04 | .652 | .01 | .02 | -.03-.05 | .50 | .617 | .01 | .02 | -.02-.05 | .05 | .467 |
|  | Loneliness |  |  |  |  |  | -.13 | .20 | -.26-.52 | -.06 | .518 | .22 | .19 | -.16-.59 | .10 | .258 | .25 | .18 | -.11-.61 | .11 | .169 |
| **Change** | **Anxiety change** |  |  |  |  |  |  |  |  |  |  | **-.39** | **.14** | **-.67--.11** | **-.19** | **.008** | **-.42** | **.14** | **-.70--.15** | **-.21** | **.002** |
|  | Depression change |  |  |  |  |  |  |  |  |  |  | **-.43** | **.15** | **-.73--.14** | **-.21** | **.004** | -.26 | .15 | -.56-.04 | -.13 | .083 |
| **Current** | **Self-rated health** |  |  |  |  |  |  |  |  |  |  |  |  |  |  |  | **.58** | **.17** | **.25-.92** | **.26** | **.001** |
| Constant | | 2.41 | 1.49 | -.53-5.36 |  | .107 | 2.47 | 1.51 | -.51-5.45 |  | .103 | 4.07 | 1.47 | 1.17-6.98 |  | .006 | 3.25 | 1.42 | .43-6.06 |  | .024 |
| r2 change | | .361 | | | | | .004 | | | | | .083 | | | | | .052 | | | | |
| Adjusted r2 | | .356 | | | | | .349 | | | | | .424 | | | | | .474 | | | | |

B = unstandardised coefficient β = standardised coefficient ADL = Activities of Daily Living (measured by Townsend Functional Ability Scale). IPIP ES = IPIP Emotional Stability

In bold = significant after FDR calculation.

Table S3: hierarchical regression for QoL social domain wave 4

| Category | Predictor | B | SE | 95% CI for B | β | p |
| --- | --- | --- | --- | --- | --- | --- |
| **Baseline QoL** | **Social domain w1** | **.53** | **.09** | **.34-.71** | **.46** | **<.001** |
| Constant | | 8.32 | 1.64 | 5.08-11.56 |  | <.001 |
| r2 change | | .209 | | | | |
| Adjusted r2 | | .203 | | | | |

B = unstandardised coefficient β = standardised coefficient

In bold = significant after FDR calculation.

Table S4: hierarchical regression for QoL environment domain wave 4

| Category | Predictor | B | SE | 95% CI for B | β | p | B | SE | 95% CI for B | β | p | B | SE | 95% CI for B | β | p |
| --- | --- | --- | --- | --- | --- | --- | --- | --- | --- | --- | --- | --- | --- | --- | --- | --- |
| **Baseline QoL** | **Envir. domain w1** | **.50** | **.08** | **.34-.65** | **.50** | **<.001** | **.40** | **.09** | **.21-.58** | **.40** | **<.001** | **.32** | **.09** | **.14-.51** | **.33** | **.001** |
| Baseline | IPIP ES |  |  |  |  |  | .02 | .02 | -.01-.06 | .11 | .187 | .02 | .02 | -.02-.06 | .09 | .293 |
|  | Loneliness |  |  |  |  |  | -.21 | .16 | -.11-.53 | -.11 | .205 | -.16 | .16 | -.15-.47 | -.09 | .313 |
|  | Social class |  |  |  |  |  | -.19 | .17 | -.51-.14 | -.10 | .260 | -.25 | .16 | -.56-.06 | -.14 | .119 |
| Current | Anxiety |  |  |  |  |  |  |  |  |  |  | -.06 | .05 | -.16-.04 | -.10 | .245 |
|  | Depression |  |  |  |  |  |  |  |  |  |  | -.06 | .06 | -.19-.06 | -.09 | .331 |
|  | ADL |  |  |  |  |  |  |  |  |  |  | -.05 | .03 | -.11-.01 | -.14 | .098 |
|  | Self-rated health |  |  |  |  |  |  |  |  |  |  | .31 | .16 | -.00-.61 | .16 | .050 |
| Constant | | 7.51 | 1.33 | 4.87-10.16 |  | <.001 | 7.81 | 1.63 | 4.59-11.04 |  | <.001 | 9.21 | 1.93 | 5.38-13.04 |  | <.001 |
| r2 change | | .254 | | | | | .031 | | | | | .092 | | | | |
| Adjusted r2 | | .248 | | | | | .260 | | | | | .332 | | | | |

B = unstandardised coefficient β = standardised coefficient ADL = Activities of Daily Living (measured by Townsend Functional Ability Scale). IPIP ES = IPIP Emotional Stability

In bold = significant after FDR calculation.

Table S5: hierarchical ordinal regression for QoL item wave 4

| Category | Predictor | Est-imate | SE | p | OR | 95% CI for OR | Est-imate | SE | p | OR | 95% CI for OR | Est-imate | SE | p | OR | 95% CI for OR | Est-imate | SE | p | OR | 95% CI for OR |
| --- | --- | --- | --- | --- | --- | --- | --- | --- | --- | --- | --- | --- | --- | --- | --- | --- | --- | --- | --- | --- | --- |
| **Baseline QoL** | QoL item w1 | **1.38** | **.35** | **<.001** | **3.97** | **1.99-7.92** | **1.11** | **.37** | **.003** | **3.03** | **1.47-6.24** | **1.09** | **.39** | **.005** | **2.98** | **1.40-6.35** | .32 | .48 | .503 | 1.38 | .54-3.55 |
| Baseline | **Loneliness** |  |  |  |  |  | -.73 | .23 | .002 | .48 | .31-.76 | **-.79** | **.25** | **.001** | **.45** | **.28-.73** | **-.91** | **.31** | **.004** | **.40** | **.22-.74** |
| Change | ADL change |  |  |  |  |  |  |  |  |  |  | -.48 | .22 | .029 | .62 | .40-.95 | -.34 | .30 | .246 | .71 | .40-1.27 |
|  | Depression change |  |  |  |  |  |  |  |  |  |  | -.48 | .23 | .036 | .62 | .40-.97 | -.32 | .29 | .277 | .73 | .41-1.29 |
| Current | Lung function |  |  |  |  |  |  |  |  |  |  |  |  |  |  |  | .30 | .25 | .230 | 1.35 | .83-2.21 |
|  | **Self-rated health** |  |  |  |  |  |  |  |  |  |  |  |  |  |  |  | **1.05** | **.38** | **.005** | **2.85** | **1.37-5.94** |

OR = Odds Ratio for one point increase in predictor. ADL = Activities of Daily Living (measured by Townsend Functional Ability Scale).

In bold = significant after FDR calculation.

Table S6: hierarchical ordinal regression for health QoL item wave 4

| Category | Predictor | Est-imate | SE | p | OR | 95% CI for OR | Est-imate | SE | p | OR | 95% CI for OR | Est-imate | SE | p | OR | 95% CI for OR |
| --- | --- | --- | --- | --- | --- | --- | --- | --- | --- | --- | --- | --- | --- | --- | --- | --- |
| **Baseline QoL** | **Health QoL item w1** | **1.22** | **.24** | **<.001** | **3.40** | **2.12-5.46** | **1.19** | **.24** | **<.001** | **3.30** | **2.05-5.31** | **.88** | **.26** | **.001** | **2.40** | **1.46-3.96** |
| Change | ADL change |  |  |  |  |  | -.37 | .19 | .052 | .69 | .46-1.00 | -.35 | .20 | .074 | .70 | .48-1.03 |
|  | Depression change |  |  |  |  |  | -.26 | .19 | .247 | .80 | .55-1.17 | .14 | .21 | .509 | 1.15 | .76-1.73 |
| **Current** | **Self-rated health** |  |  |  |  |  |  |  |  |  |  | **1.27** | **.27** | **<.001** | **3.76** | **2.12-6.03** |

OR = Odds Ratio for one point increase in predictor. ADL = Activities of Daily Living (measured by Townsend Functional Ability Scale).

In bold = significant after FDR calculation.
